# Does government food demonstration intervention influence household dietary diversity in the Upper West Region of Ghana?

**DOI:** 10.1371/journal.pone.0302869

**Published:** 2024-05-08

**Authors:** Cornelius K. A. Pienaah, Sulemana Ansumah Saaka, Herwin Ziemeh Yengnone, Mildred Naamwintome Molle, Isaac Luginaah

**Affiliations:** Department of Geography and Environment, University of Western Ontario, London, Ontario, Canada; SDD - University of Business and Integrated Development Studies, GHANA

## Abstract

Dietary diversity is crucial in ensuring food and nutrition security. In low-middle-income countries, people frequently prioritize the quantity of food they consume over its quality due to a lack of availability and financial limitations. As a result, achieving dietary diversity is often overlooked in favor of ensuring adequate caloric intake. Through a social cognitive theory perspective, our study examines the relationship between food demonstrations and household dietary diversity in Ghana’s Upper West Region utilizing cross-sectional survey data from 517 smallholder farmer households. The results from ordered logistic regression presented in odds ratio (OR) show that participating in food demonstrations (OR: 2.585, p<0.01), engaging in home gardening (OR: 1.932, p<0.001), having access to credit (OR: 1.609, p<0.01), self-rated good nutritional status (OR: 1.747, p<0.01), and Waala ethnicity (OR: 3.686, p<0.001) were all positively associated with high household dietary diversity. Conversely, living in the Wa West district was associated with lower dietary diversity (OR: 0.326, p<0.001). Our research findings suggest that policymakers may want to consider implementing community-based educational programs, such as home and school visits for food demonstrations and sensitizations, promoting mother-to-mother support groups for dietary diversity education, nutrition counseling services, and using role-play and local media. In addition, strengthening local agricultural policies through food banks, indigenous seed development, and mobile food markets and enhancing public-private partnerships like the Ghana Schools Feeding Programme and National Food Buffer Stock company could improve the supply chain and distribution networks for diverse food items. Implementing these interventions in the Upper West Region of Ghana could improve health, well-being, food security, and nutritional outcomes.

## 1. Introduction

Household dietary diversity (HHD) encompasses the range and variety of foods or food groups a household consumes within a specific time frame [1,2]. It is a crucial indicator of the quality of a person’s diet and an essential component of maintaining overall nutritional well-being in the context of increasing global food insecurity [3]. The global concern for prioritizing dietary diversity has become more pressing as countries face undernutrition, micronutrient deficiencies, and over-nutrition [2,4]. These challenges underscore the nature of health and socio-economic development threats [4]. Despite Sub-Saharan Africa’s relatively diverse food resources, many populations, including those in Ghana’s Upper West Region (UWR), still suffer from nutritional deficiencies and food insecurity [5–9]. Women and children are the most affected by poor nutrition and suffer from severe related health consequences in the UWR [10]. According to the 2022 Ghana Demographic and Health Survey, 18% of children under 5 are stunted, 6% are wasted, and 12% are underweight. Additionally, 2% of children under 5 are overweight [11]. In the UWR of Ghana, about 25.11% of children under the age of five experience stunting, while 7.31% suffer from wasting [12]. Furthermore, a significant proportion (63.6%) of the region’s population faces food insecurity, highlighting its distinct challenges [13]. The heightened food insecurity in UWR has had several negative consequences, such as undernutrition, micronutrient deficiencies, and nutrition-related non-communicable diseases [6,8,9,13]. Pregnant women and young children are more vulnerable to these effects, raising concerns about residents’ overall health and nutrition [6]. The experience of food insecurity in UWR has resulted in a need for more dietary diversity [14,15]. Most residents rely on a few food items lacking essential minerals and vitamins for their overall well-being [11,14]. These meals are usually composed of starchy staples such as maize and millet. They are deficient in proteins, vegetables, and fruits, resulting in malnutrition, stunted growth, and other health concerns [6,14,16].

In recognition of the poor nutritional status of the people in the UWR, the Women in Agriculture Development (WIAD) Unit under the Department of Agriculture (DOA), the Ghana Health Service (GHS), and multiple collaborated non-governmental organizations (NGOs) embarked on an intervention of food demonstration (FD) to improve malnutrition, achieve food security, and promote healthy living through dietary diversity towards the UWR’s food future. Food demonstrations (FDs) are community-based presentations that showcase food preparation and consumption techniques using locally available ingredients sourced from the area (such as shea butter, moringa leaves, orange-fleshed sweet potatoes, lemon, honey, cow milk, baobab leaves and pulp powder, “dawadawa” cake and pulp powder, soybeans, “egusi,” “sara,” “jongboro,” “katuo,” cowpea, sorghum, rice, millet, maize, yam, Bambara beans, groundnuts, egg, fish, and poultry products). These demonstrations provide practical tools for education, inspiration, and behavior change in the community. FDs are held in community centers or local schools where community members, especially women, young mothers, and girls, come together to learn and share food preparation skills. During the demonstration session, a trained facilitator demonstrates cleaning and preparation techniques to ensure hygiene and preserve the benefits of the ingredients. The facilitator shows how to combine different ingredients to maximize their nutritional value. For example, they suggest pairing foods high in vitamin C, such as lemon or baobab fruit pulp powder, with those high in iron, such as moringa leaf powder [17]. Doing so can increase iron absorption and improve nutritional content in meals like porridge. The cooking process is interactive, and participants get hands-on experience in preparing the meals (for example, participants exchange recipes and taste-test foods like doughnuts prepared from soybean, dawadada pulp powder, and orange-fleshed sweet potatoes) for improvement. The facilitator also addresses misconceptions and uncertainties about diet and offers tips while promoting experience and new knowledge from participants on creating diverse and well-balanced meals. The sessions promote nutritional awareness, encourage the utilization of local food resources, empower communities to make healthier food choices, and encourage them to revamp overlooked indigenous foods. During FD sessions, farmers are encouraged to diversify crop cultivation and livestock production to cater to their dietary needs.

Nevertheless, studies on FD are relatively scarce in SSA, especially in northern Ghana. Many scholars have reported on food security and dietary diversity experiences [8,9,13,18–20], neglecting how FDs translate into HDD. Despite the intervention of FD, it remains to be seen whether these demonstrations translate into adequate dietary diversity at the household level, considering the socio-economic, socio-cultural, resource-poor, and environmental dynamics in the UWR of Ghana. Given this context, this study investigates the effectiveness of FDs in promoting dietary diversity among households. We hypothesized that FDs have a positive impact on dietary diversity.

## 2. Theoretical framework: Social cognitive theory

Albert Bandura developed Social Cognitive Theory (SCT) from Social Learning Theory (SLT) in the 1960s [21]. It evolved into the SCT in 1986, proposing that learning occurs in a social context, with a dynamic and reciprocal interplay of the person, environment, and behavior [22]. The focus on the social impact of external and internal social reinforcement distinguishes SCT [23]. SCT considers how people learn and retain behavior and the social setting in which individuals do the behavior. The theory finds a person’s prior experiences, which influence whether behavioral activity will occur [22]. These previous experiences form reinforcing expectations and expectancies, all determining whether a person will participate in each behavior and the reasons for that behavior [21,23].

According to SCT, health-related behavior, such as food choices, is a constant reciprocal interplay between persons, their surroundings, and their activities [22]. The concept that people learn not just via their own experiences but also by seeing the behaviors and outcomes of others is central to SCT. Individual cognitive processes mediate this learning and may be modified or impacted by external social cues such as food demonstrations. SCT offers an essential lens for analysis in the context of our study domain, “Does food demonstration improve household dietary diversity in the Upper West Region of Ghana?”. Food demonstrations may be seen as a social and environmental aspect encouraging observational learning. These demonstrations expose households to new food preparation skills, nutritional information, and various dietary alternatives. Household members, especially those purchasing food and preparation, become agents in their health promotion, absorbing new information and possibly applying it to their food choices [24]. SCT enables us to investigate whether dietary diversity improves after participation in food demonstrates and the cognitive and social mechanisms that may account for this shift.

## 3. Materials and methods

### 3.1 Study context

**Fig 1:**
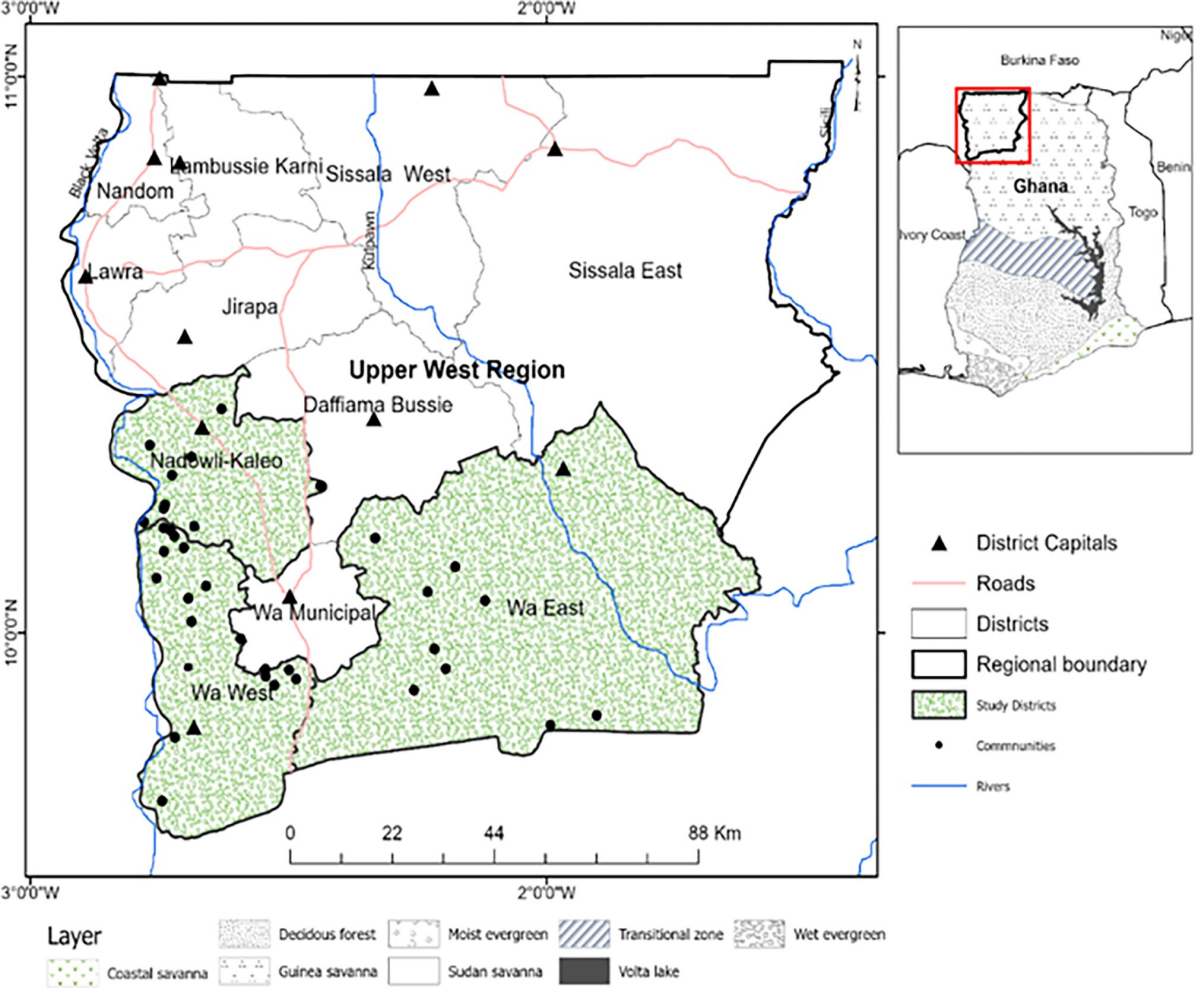
Modified Map of Upper West Region showing the study area.

The research was conducted in northern Ghana’s Upper West Region (UWR). The region has a landmass of 18,476 km square and spans longitudes 1⁰ 36’ to 3⁰ West and latitudes 9⁰ 48’ to 11⁰ North, as seen in Fig 1 [25]. The region has a population of 901,502 and borders Burkina Faso, Ghana’s Upper East Region, and the Savannah Region to the north, east, and west respectively [26]. The UWR is characterized by bushfires and harmattan in the dry season. The region has drought-resistant trees like Adansonia digitata (baobab), Blighia sapida (ackee), dawadawa (Parkia biglobosa), Shea (Vitellaria paradoxa), and Acacia albida (Faidherbia albida) [27,28]. Subsistence agriculture is the main economic activity, employing around 80% of the population and about 80.4% of households in the region, and women constitute 42% [25,27–29]. Most women in the UWR rely on shea products for livelihoods [27]. The UWR has a Sudanian climate, unpredictable temperatures, and annual precipitation ranging from 840mm to 1,400mm [25,28,30]. The region faces significant challenges in ensuring fair and sustainable access to water [30]. The environment is semi-arid and prone to multiple and severe climate stressors that have a substantial impact on agricultural productivity [30], poor health [9,31], out-migration [9], and environmental pollution [31]. Food security and hunger are particularly pressing issues in the UWR [8,9,13,18–20]. The UWR is one of the poorest in the country. Shockingly, according to statistics from the Ghana Statistical Service in 2019, 90% of the population, equivalent to 9 out of every 10 people, survive on less than one dollar per day [32]. In the districts of Wa East, Wa West, and Nadowli-Kaleo, where the study was conducted, the poverty rates are as high as 92.4%, 83.8%, and 68.5%, respectively [33].

The levels of malnutrition in the UWR are relatively high, as per a study conducted in Northern Ghana that included this region. The study found that children in these areas have a considerable prevalence of malnutrition, with 27.2%, 17.6%, and 8.2% of them experiencing stunting, underweight, and wasting, respectively [34]. Despite national efforts to address malnutrition in the northern regions of Ghana, including the Upper West, this issue remains a significant challenge. In 2012, 18.4% of children aged 0–59 months in the Northern, Upper East, and Upper West regions were underweighted, 11% were experiencing wasting, and 36.1% were stunted. These rates are significantly higher than the national averages of 11% underweight, 19% stunting, and 5% wasting [35]. A significant proportion of children under the age of five in the UWR suffer from acute malnutrition, highlighting the severity of the situation and the need for collaborative efforts to promote good nutritional practices in the region [11]. Although there have been appreciable reductions in malnutrition rates nationally, many people in Ghana, especially women and children, still suffer from stunting, emerging issues of over-nutrition, and micro-nutrient deficiencies [36]. This uneven progress reflects wide socio-economic and geographical disparities across the country, indicating that interventions have yet to be implemented uniformly or sustainably in all regions [25,28].

A study conducted by Mohsen et al. found that educational literacy and attainment have a positive impact on households’ decisions regarding food production and consumption habits, as well as on dietary diversity and food security [37]. In the 2021 Population and Housing Census (PHC) General Report, the literacy rate among the population aged six years and older in nine out of the 16 regions in Ghana is below the national figure of 69.8% [26]. However, the UWR boasts a literacy rate of 74.1%, which is higher than the national average. The Greater Accra Region has the highest literacy rate at 87.9%, while the Savannah Region records the lowest at 32.8% [26]. Although the UWR’s literacy rate falls short of the highest rates observed in regions like Greater Accra, it is still an impressive accomplishment. Out of the population in the UWR, only 25.9% are not literate, indicating that a significant portion of the population possesses basic literacy skills [24]. This could influence households’ dietary diversity. Regardless of the illiteracy rate in the UWR, Nadowli-Kaleo, Wa East, and Wa West significantly improve literacy levels, respectively [26].

The UWR has a unique blend of rich cultural and nutritional heritage, agricultural reliance, and dietary challenges. With vast rural landscapes, the region’s diet centers around staple cereals like maize, millet, and sorghum, occasionally complemented by legumes such as groundnut and cowpeas. However, while these staples are essential, they often overshadow the full range of nutrients required for optimal health. Household dietary diversity in the UWR is cyclical, thriving during the rainy season with a wealth of fresh produce but dwindling during the dry season due to limited availability. Economic constraints further limit dietary options, with households prioritizing calorie-dense staples over potentially pricier, diverse foods [25,34]. This, combined with little awareness and access to the region’s nutritious indigenous foods, has contributed to nutritional deficiencies, particularly in children and women [6,25,38]. As modernization advances, processed foods are gaining popularity, introducing diversity but not necessarily improving nutrition. Cultural preferences, deeply rooted in tradition, also shape dietary habits in the UWR, sometimes neglecting nutrient-rich foods not aligned with customary practices [25,33,38,39]. Thus, while the UWR boasts agricultural potential, balancing tradition, economic realities, and the need for dietary diversity remains a pressing challenge.

### 3.2 Data and sample

This study is part of a larger project on *“the impact of the Community Resource Management Area (CREMA) on improving livelihoods and climate change resilience in UWR*, *Ghana*.*”* Data was collected using a questionnaire between 10 November 2022 and 31 January 2023 from 517 households involved in agriculture, based on the criteria specified in the 2017/18 Census of Agriculture by the Ghana Statistical Services [25]. An agricultural household is defined as one where at least one member is involved in farming. The study was conducted in two phases. Firstly, 36 communities were selected from three districts. Wa East, Wa West, and Nadowli-Kaleo districts were chosen for this study because they have implemented Community Resource Management Areas (CREMAs) and are part of the broader study project. CREMAs empower local communities to govern and manage natural resources [40]. CREMAs involve the establishment of a Community Resource Management Committee (CRMC) and CREMA Executive Committee (CEC), drafting a constitution and bylaws, and managing natural resources. The government awards the CREMA a certificate of devolution, granting the community legal power to manage their natural resources sustainably. CREMAs aim to restore degraded ecosystems, raise environmental awareness, and support farmers in improving their agricultural practices. Individuals, organizations, and companies can contribute to the conservation fund by signing conservation agreements to support CREMA activities [27,30,40].

Secondly, every fifth household from the listed households in these communities was chosen. We obtained verbal consent from all research participants (the primary representative of the household). Verbal consent was chosen based on the participants’ literacy levels and cultural preferences in the research area and region. To capture this verbal agreement, we sought the participants’ consent in the presence of a spouse or other adult household member and a local community opinion leader (like a CREMA executive, assembly member, or unit committee member) knowledgeable of the study’s objectives and ethical implications. All verbal consents were based on their agreement of either a “yes” or “no” before the interview. Only participants who consented with a yes were interviewed. Our research did not include minors; all participants (in most cases, the primary farmers or agriculturalists) were adults aged 18 or older who participated in the survey on behalf of their respective households. Participation in this study was voluntary, and all ethical considerations were met through obtaining formal verbal consent. We obtained ethical approval for this study from the Non-Medical Research Ethics Board (NMREB) of the University of Western Ontario, Canada. Information on various topics related to the farmers and their households was also collected, including demographics, socioeconomic factors, sociocultural characteristics, dietary diversity, food, water, energy, nutrition securities, climate change adaptation, mitigation, preparedness, and resilience.

### 3.3 Measures

Household food security includes dietary diversification [41–43]. The outcome variable for this is “Household Dietary Diversity.” This study measured Household Dietary Diversity (HDD) using the 12-point number of food groups [43] consumed in the past week. The outcome variable question from the dietary assessment was, "What were the dietary intake patterns, in terms of meals and foods, of you or your household during the previous week, both during the day and at night?" Each food category [A. Cereals, B. Root and tubers, C. Vegetables, D. Fruits, E. Meat, poultry, offal, F. Eggs, G. Fish and seafood, H. Pulses/legumes/nuts, I. Milk and milk products, J. Oil/fats, K. Sugar/honey, and L. Miscellaneous] ingested in the prior week was worth one point, giving each household a maximum dietary variety score of 12 points. We then developed a Household Dietary Diversity Score (HDDS) [43]. The HDDS was classified as low, medium, or high based on the number of food categories eaten by the household: ≤3, 4–6, or 7+ [2,43]. We then coded HHD as (0 = low dietary diversity, 1 = medium dietary diversity, 2 = high dietary diversity). Access to diverse food groups rises with HDDS, so we predicted high dietary diversity. Since our research focused on dietary quality rather than overall calorie intake, we employed the HDDS that accounts for diversified food consumption. The indicator provides a more detailed view of nutritional quality than calorie consumption, which measures energy intake. Other authors have used this categorization [44,45].

Based on the underpinning of social cognitive theory, we constructed food demonstration as the focal independent variable. We asked participants if they or any household member had participated in a food demonstration in the past 12 months. We coded their responses as either "no" (0) or "yes" (1). This allowed us to predict the impact of food demonstration on high dietary diversity. From the social cognitive theory perspective, we also included theoretically relevant variables based on broader food and nutrition security literature [8,9,13,18–20,30,46–48]. These include home gardening (no = 0, 1 = yes), livestock rearing (no = 0, 1 = yes), access to credit (no = 0, 1 = yes), receipt remittances (no = 0, 1 = yes), infants (0 = no under five children, 1 = under five children present), and self-rated nutrition status (based on self-rating of entire household), The choices were "Excellent," "Very good," "Good," "Fair," and "Poor." Following previous research [30,31,49], we divided the responses into two categories: poor nutrition (including "Fair" and "Poor") and good nutrition (including "Excellent," "Very Good," and "Good") coded as (0 = poor nutrition, 1 = good nutrition), gender (0 = male, 1 = female), educational level (0 = no formal, 1 = primary, 2 = secondary and above), age (0 = 18–29, 1 = 30–39, 2 = 40–49, 3 = 50–59, 4 = 60+), marital status (0 = married, 1 = single, 2 = divorced/widowed); religion (0 = Christian, 1 = Muslim, 2 = African tradition), ethnicity (0 = Dagaaba, 1 = Sissala, 2 = Brifo, 3 = Waala), household size (0 = 1–4, 1 = 5–8, 2 = 9 and above), wealth (0 = poorest, 1 = poorer, 2 = middle, 3 = richer, 4 = richest). Ownership of agricultural land was added as a continuous variable. Finally, the district of residence was coded as (0 = Nadowli-Kaleo, 1 = Wa East, 2 = Wa West).

### 3.4 Analytical approach

Following univariate analysis to explore the descriptive function of the data, the proportional odds logistic regression model was used to investigate the association between food demonstration and household dietary diversity at the bivariate and multivariate levels since the "household dietary diversity" outcome variable was ordered [50]. We controlled for demographic, household, and environmental factors at the multiple-ordered logistic regression level. The following is the logistic regression model’s computation equation [51].


$$log\frac{P(Yij\leq 1)}{\left(1-P(Yij\leq 1\right)}=a_{0}+\sum_{k=1}^{p-1}\left(a_{jk}X_{\mathrm{ijk}}+V_{ij,}C=1,\ldots .\Omega -1\right)$$

Where *P* (*Yij* ≤ 1) denotes the likelihood that an event will occur and (1—*P*(*Yij* ≤ 1) represents the likelihood that the event will not happen, *Xijk* = explanatory variables, where (k = 1) is the first and (p—1) is the final explanatory variable. In the logistic model, Vij is the error term, *α*_0_ and Ω - 1 are the intercept terms, and *α*_*jk*_ is the coefficient term [51]. The regression coefficients are shown as odds ratios; odds ratios more than one (OR > 1) indicate a greater chance of having a high dietary diversity, while odds ratios less than one (OR < 1) indicate a lower likelihood of having a dietary diversity [50,51]. We determined multicollinearity among predictor variables using the Variance Inflation Factor. A VIF score below 10 implies no multicollinearity, whereas over ten shows multicollinearity and requires variable removal from the model. Our VIF study found no multicollinearity. Additionally, we used the Brant test to verify that the ordinal logistic regression model satisfied proportional odds [50,51]. All data analysis was completed using Stata version 18.0.

## 4. Results

### 4.1 Univariate results

The univariate results are shown in Table 1. The study reveals that the average household dietary diversity score is 6.44, with low diversity at 11.03%, moderate diversity at 43.33%, and high diversity at 45.65%. Food demonstrations are absent in 88.97% of households. Also, home gardening is prevalent in 27.66% of households. Livestock raising is practiced in 79.50% of households. Access to credit is higher in 40.23% of households, while remittances are received in 12.19% of households. Children under five live in 64.99% of households. Self-rated nutrition status is considered poor by 61.90% of households.

**Table 1:** Descriptive analysis of the sample.

Variables	Percentage (%)	Frequency
**Household dietary diversity score (HDDS)**	6.44 (mean), SD: 2.41	Min = 1, Max = 12
Low	11.03	57
Moderate	43.33	224
High	45.65	236
**Food demonstration**		
No	88.97	460
Yes	11.03	57
**Home Gardening**		
No	72.34	374
Yes	27.66	143
**Livestock rearing**		
No	20.50	106
Yes	79.50	411
**Access to credit**		
No	59.77	309
Yes	40.23	208
**Receipt remittances**		
No	87.81	454
Yes	12.19	63
**Infant children**		
No under-five children	35.01	181
Under five children present	64.99	336
**Self-rated nutrition status**		
Poor	61.90	320
Good	38.10	197
**Gender**		
Male	62.86	325
Female	37.14	192
**Level of education**		
No formal education	71.95	372
Primary	18.57	96
Secondary or higher	9.48	49
**Age of respondents**		
18–29	19.15	99
30–39	18.76	97
40–49	26.31	136
50–59	19.73	102
60+	16.06	83
**Marital status**		
Single	10.83	56
Married	77.37	400
Divorced/Widowed	11.80	61
**Religion**		
Christian	55.51	287
Muslim	29.79	154
African Tradition	14.70	76
**Ethnicity**		
Dagaaba	60.54	313
Sissala	15.28	79
Brifo	12.38	64
Waala	11.80	61
**Household size**		
1–4	26.89	139
5–8	43.71	226
9+	29.40	152
**Household wealth**		
Poorest	24.95	129
Poorer	16.63	86
Middle	19.92	103
Richer	17.60	91
Richest	20.89	108
**Land size (continuous)**	50.76(mean), SD: 206.57	Min = 0, Max = 2000
**Districts of residence**		
Nadowli-Kaleo	23.40	121
Wa East	32.30	167
Wa West	44.29	229

### 4.2 Bivariate results

The study’s bivariate ordered logistic regression results, presented in Table 2, reveal several significant factors that positively impact household dietary diversity. Households participating in food demonstrations (OR:3.510; p<0.001) were associated with reporting high dietary diversity compared to those not participating. Households that engaged in home gardening (OR:2.739; p<0.001), livestock rearing (OR:1.559; p<0.01) or had access to credit (OR:1.533; p<0.001) were more associated with high dietary diversity compared to those not into any of these activities. The study also found that households with young children (under 5) (OR:1.659; p<0.001) and good self-rated nutritional status (OR:1.978; p<0.001) had a higher likelihood of high dietary diversity compared to households without infants (under-five children) and poorly rated nutrition. Individuals with primary education (OR:1.647; p<0.01) or secondary education (OR:1.803, p<0.05) have a greater likelihood of achieving high dietary diversity compared to those with no formal education. Conversely, older household members, those aged 40–49, 50–59, and 60 years and above, have lower odds of having high dietary diversity compared to younger household members. However, individuals affiliated with the Muslim faith (OR:2.675; p<0.001) compared to the Christian faith and belonging to the Sissala (OR:4.911; p<0.001) and Waala (OR:2.826; p<0.001) ethnic groups compared to Dagaaba were positively associated with high dietary diversity. On the other hand, those belonging to the Brifo ethnic group (OR: 0.271; p<0.001) are less likely to have high dietary diversity. It is worth noting that households in the Wa West district have lower odds of achieving high dietary diversity (OR: 0.374; p<0.001) compared to the Nadowli-Kaleo district.

**Table 2:** Ordered logistic regression analysis of food demonstration and household dietary diversity.

Variables	Bivariate OR(SE)	[95% CI]	Multivariate OR(SE)	[95% CI]
**Food demonstration** (ref: No)				
Yes	3.510(1.107)***	1.891 6.516	2.585(1.137)**	1.091 6.124
**Home gardening** (ref: No)				
Yes	2.739(0.546)***	1.852 4.050	1.932(0.472)***	1.197 3.119
**Livestock rearing** (ref: No)				
Yes	1.559(0.331)**	1.028 2.364	1.387(0.384)	0.806 2.386
**Access to credit** (ref: No)				
Yes	1.533(0.264)***	1.093 2.151	1.609(0.380)**	1.012 2.557
**Receipt Remittances** (ref: No)				
Yes	1.282(0.341)	0.760 2.162	0.660(0.221)	0.342 1.273
**Infants Children** (ref: No under five child)				
Children under five present	1.659(0.293)***	1.173 2.346	1.341(0.332)	0.826 2.179
**Self-rated nutrition status** (ref: Poor)				
Good	1.978(0.351)***	1.396 2.802	1.747(0.447)**	1.057 2.887
**Gender** (ref: Male)				
Female	0.764(0.132)	0.543 1.0748	0.752(0.198)	0.449 1.262
**Level of education** (ref: No formal education)				
Primary	1.647(0.373)**	1.056 2.567	1.106(0.360)	0.584 2.094
Secondary or higher	1.803(0.541)*	1.001 3.249	1.592(0.691)	0.680 3.728
**Age of respondents** (ref: 18–29 years)				
30–39	0.866(0.242)	0.500 1.498	0.937(0.390)	0.414 2.118
40–49	0.583(0.1504)**	0.352 0.967	0.615(0.239)	0.287 1.319
50–59	0.466(0.127)***	0.273 0.797	0.578(0.244)	0.252 1.324
60+	0.487(0.143)***	0.274 0.866	0.493(0.225)	0.202 1.206
**Marital status** (ref: Single)				
Married	1.048(0.2877)	0.611 1.795	1.290(0.525)	0.581 2.865
Divorced/Widowed	0.759(0.269)	0.378 1.522	1.559(0.799)	0.570 4.259
**Religion** (ref: Christian)				
Muslim	2.675(0.539)***	1.801 3.973	0.982(0.321)	0.516 1.865
African Tradition	0.624(0.159)	0.378 1.030	0.772(0.2350	0.425 1.402
**Ethnicity** (ref. Dagaaba)				
Sissala	4.911(1.397)***	2.811 8.577	1.872(0.951)	0.691 5.066
Brifo	0.271(0.078)***	0.153 0.477	0.670(0.257)	0.316 1.421
Waala	2.826(0.803)***	1.618 4.934	3.686(1.595)***	1.578 8.609
**Household size** (ref: 1–4)				
5–8	0.944(0.193)	0.631 1.411	0.863(0.244)	0.496 1.502
9+	0.947(0.214)	0.607 1.476	0.738(0.245)	0.384 1.417
**Household wealth** (ref: Poorest)				
Poorer	1.073(0.285)	0.637 1.806	0.762(0.256)	0.394 1.475
Middle	1.338(0.338)	0.814 2.197	0.971(0.312)	0.517 1.826
Richer	1.166(0.305)	0.698 1.948	0.674(0.235)	0.339 1.338
Richest	2.420(0.626)**	1.457 4.018	0.986(0.352)	0.489 1.987
**Land size** (continuous)	0.002(0.001)	-0.005 0.001	1.000(0.001)	0.999 1.001
**Districts of residence** (ref: Nadowli-Kaleo)				
Wa East	2.213(0.533)***	1.380 3.548	1.332(0.570)	0.575 3.085
Wa West	0.374(0.083)***	0.242 0.578	0.326(0.101)***	0.177 0.600
**LR chi2 (30)**			148.47 0.1750 -349.875	
**Pseudo R2**				
**Log-likelihood**				

P < 0.1

*P < 0.05

**P < 0.01

***P < 0.001: SE (standard error), CI (Confidence Interval), OR (Odds ratio).

### 4.3 Multiple regression analysis results

Table 2 shows the results of a multiple-ordered logistic regression analysis. Several variables significantly impacted dietary diversity. The chances of having a more diversified diet increased significantly with households’ participation in food demonstrations (OR: 2.585; p<0.01) compared to households not participating in FDs. Households with home gardening had nearly twice the probability of having diversified diets (OR: 1.932; p<0.001) compared to households that do not have. Access to credit also played a significant role in enhancing dietary diversity probabilities. Households with access to credit were about two times more likely to have high dietary diversity (OR: 1.609; p<0.01) than those without. Individuals who rated themselves healthier in nutrition had a more diversified diet (OR: 1.747; p<0.01) compared to those who rated their nutritional status as poor. Among ethnic groups, the Waala ethnicity had three times substantially impacted the chances of achieving a diversified diet (OR: 3.686; p<0.001) compared to the Dagaaba group. However, residents in the Wa West district had a considerably lower likelihood of achieving high dietary diversification (OR: 0.326; p<0.001) than those in the Nadowli-Kaleo district.

## 5. Discussion

The findings of this research align closely with the fundamental tenets of Social Cognitive Theory. The study demonstrates that combining individual and environmental factors significantly influences high dietary diversity. This emphasizes the importance of implementing comprehensive interventions to foster healthier and more varied eating habits. This research contributes to our theoretical understanding of dietary diversity. Using SCT as a framework for knowledge utilization interventions could serve as a valuable basis for future research and development.

Consistent with the study hypothesis, our findings demonstrate a positive association between participation in food demonstrations and high dietary diversity. This highlights the potential benefits of hands-on educational interventions in improving household eating habits. The profound influence is comprehended by considering the focus placed by SCT on observational learning and self-efficacy [21,22]. Food demonstrations serve as an environmental stimulus that augments individuals’ capacity to make a wide range of food selections by offering indirect experiences and possibilities for observational learning. In essence, seeing the process of preparing healthy meals positively impacts an individual’s self-assurance and ability to recreate comparable dishes in their kitchen. Consistent empirical evidence from rural Senegal has suggested that women who participated in nutrition education and FDs and nutrition education reported higher dietary diversity in the households than those who did not [52].

Invariably, households that engaged in home gardening activities were positively associated with high dietary diversification. This implies that households with the resources and expertise to cultivate a variety of foods are more likely to include them in their meals, resulting in a more nutritious intake. From the perspective of SCT, home gardening may boost self-efficacy in one’s capacity to generate one’s food, which in turn influences dietary choices. In this manner, the garden is transformed into an environmental component that fosters a feeling of agency and self-regulation in food patterns [23,53–57]. This finding backs up a previous study that showed that home gardening increases household food security and diversifies diets in rural Bangladesh, Myanmar, Panama, and Ethiopia by offering access to various vegetables and fruits [54–57]. Given the Upper West Region’s agricultural environment, home gardening is a practical and culturally appropriate option for dietary diversification and food diversity.

Likewise, access to credit was found to be positively associated with high dietary diversity. This suggests that financial inclusion may increase the range of foods households can purchase, leading to a more enriched and diverse diet. Within the context of the SCT, the availability of financial resources may be seen as an environmental determinant that either facilitates or restricts an individual’s agency [21–24]. There are several potential explanations for our findings. For example, access to credit can provide rural households with the means to invest in their agricultural activities, enabling them to purchase improved planting seeds, farm tools, equipment, or fertilizers, pay farm labor and tractor services, and invest in post-harvest management. This can increase the quantity and quality of food produced, improving the households’ diet and increasing their income from selling surplus produce. Credit access can also increase entrepreneurial and off-farm activities related to food processing, storage, or selling, such as small businesses (shea butter processing and beekeeping). In times of crop failure, natural disasters (floods, droughts, dry spells, storm surges, and erratic rainfall), or health emergencies (like COVID-19 and meningitis), access to credit can be a buffer or safety net against economic uncertainties or environmental shocks. It can also contribute to dietary diversity through enhanced food storage (ability to purchase storage materials) and preservation methods (like investing in mini silos and refrigeration systems). Furthermore, with better financial stability provided by access to credit, households might invest more in education, leading to better knowledge about nutrition and the importance of dietary diversity. However, increased financial autonomy among people is associated with a heightened capacity to acquire a wide range of food items, reinforcing that establishing a conducive setting plays a crucial role in fostering the adoption of nutritious dietary practices [57,58]. Our results align with prior scholarly works in Nigeria, Ethiopia, northern Ghana, and Malawi that suggest an association between enhanced financial accessibility and enhanced food security [59–62].

Notably, a substantial association existed between self-rated nutritional status and high dietary diversity. This implies a reciprocal link where a more nutritious diet improves nutritional health self-perception or that nutritional awareness stimulates more diverse food choices. From a SCT viewpoint, the observed phenomenon may be explained by an individual’s level of self-efficacy about their dietary decisions [22–24]. Individuals who see themselves as having a good nutritional status are more inclined to participate in behaviors that promote dietary variety [63]. Bandura posits that people who strongly believe in their ability to attain high self-efficacy are inclined to participate in behaviors conducive to realizing these outcomes [64]. Within the scope of this investigation, it is plausible that people who assessed their nutritional status as good may exhibit elevated levels of self-efficacy when selecting a more comprehensive range of nutritious food options. Essentially, the conviction in one’s capacity to maintain a good nutritional status might be a psychological facilitator, leading individuals toward a more diverse and nutritionally balanced [63]. The correlation between an individual’s self-perception and behaviors highlights the cyclical pattern of personal and environmental interactions proposed by SCT [22–24]. In this theory, engaging in good behaviors strengthens one’s belief in one’s abilities (self-efficacy), and conversely, a strong sense of self-efficacy encourages the continuation of positive behaviors [53,64]. Therefore, implementing programs that enhance dietary variety in public health should include the importance of boosting individual self-efficacy in making nutritional choices.

The study’s results on the influence of ethnicity, specifically on the Waala group, may also be situated within the framework of SCT [21–23]. This suggests that cultural and traditional dietary norms significantly influence dietary variety. Ethnicity typically impacts cultural norms, practices, and environmental variables affecting behavior [65]. In the instance of the “Waala,” their much greater chances for healthy dietary variety may imply that cultural norms or traditional cuisines within this ethnic group favor healthier eating. For example, many of the Waala ethnic groups are Muslims, and consuming vegetables and fruits heightened during Ramadan, possibly contributing to high dietary diversity. The results are consistent with the study conducted by Alamirew et al. in rural Ethiopia, which concluded that cultural beliefs and ethnic backgrounds play a significant role in shaping the dietary preferences of households [66]. Additionally, the Waala ethnic group dominates petty trading in the region. The Waala women, commonly called "market women," have significant purchasing power in the UWR markets. They use this power to buy a variety of food commodities and ingredients, which they retail and store for home consumption. This is likely a contributing factor to their diverse dietary habits. The finding supports Bandura’s argument that environmental factors such as cultural context can significantly influence personal choices and practices [53]. Hence, understanding these ethnic disparities might provide valuable perspectives for customizing public health interventions to be culturally sensitive and efficacious.

The observation that people living in the Wa West district, in the Upper West Region of Ghana, have low dietary diversity is consistent with documented socioeconomic disparities. This may indicate district-specific structural or environmental issues that need focused responses, such as poverty and restricted food sources. The Ghana Statistical Service Report 2015 revealed that Wa West district in Ghana has the highest poverty incidence and depth, recording 92.4% and 59%, respectively. The report also indicated that the number of poor people in the district is 74,297 individuals living below the poverty line, the highest in the Upper West Region [33,67]. This economic struggle often limits households’ food choices, leading them to opt for staple foods instead of a diverse range of options. The financial constraints residents face may influence their dietary diversity, given the acknowledged role of socio-economic status in determining it [67,68]. Our findings align with the research conducted by Giller et al. in Mali, Ghana, Malawi, Ethiopia, Tanzania, and Uganda, revealing socioeconomic disparities’ impact on food and nutrition security [69]. The study shows that individuals from low-income backgrounds tend to have access to food options that are high in calories but low in nutrients. This is exacerbated by limited knowledge about nutrition, dietary practices, and food availability. Examining the relationship between poverty and dietary diversity underscores the significance of implementing nutritional interventions to enhance the dietary habits of the residents in Wa West district.

Nevertheless, it is crucial to recognize the limitations of the research, particularly its use of a cross-sectional design, which limits the establishment of causation. Additionally, the use of self-reported data may introduce recall biases. People may answer questions in a way that they think will be viewed positively by others, which can lead to social desirability bias. For example, men in the resource-poor context of UWR who are unable to provide for their families may want to conceal this fact. A longitudinal study would provide significant value in establishing more robust causal relationships among the investigated variables.

## 6. Conclusion and recommendations

The research provides valuable insights for policy design by highlighting various factors that influence dietary variety in Ghana’s Upper West Region. Our findings indicate that food demonstrations significantly promote dietary diversification and deserve increased investment. Additionally, the study shows that home gardening improves nutritional diversity, emphasizing the need for agricultural policies that encourage small-scale, diversified crop production at the household level. The importance of credit in enhancing financial accessibility highlights the need for specific financial tools to assist poor households in improving their dietary needs. The socio-economic situation in the Wa West district of the Upper West Region in Ghana is closely linked to the challenges faced in achieving diversity. Poverty significantly affects food choices, often leading households towards nutritious options. Interestingly, ethnic factors also come into play amidst these disparities. The Waala ethnic group appears to have a chance of achieving diversity compared to the other ethnic groups. To address this region’s low dietary diversity, a comprehensive approach is needed to improve socioeconomic conditions while acknowledging and respecting these ethnic differences. We suggest collaborating with educational authorities, local communities, and nutrition experts to create food demonstration programs in schools and homes appropriate for different age groups, respecting diverse cultures, and having educational value. These programs can also engage parents and caregivers, thus expanding their impact beyond school and home boundaries. As Ghana progresses, it is crucial to implement holistic and strategic policies that ensure all residents have access to nutritious foods regardless of gender, sex, age, economic status, religion, district, or ethnicity. This would enhance dietary diversity and improve the region’s public health. Further studies should consider reviewing FDs in Ghana to contribute to the discourse of nutrition policies in Ghana.
